# Supplementary material for: Assessment of Toxigenic Fusarium Species and Their Mycotoxins in Brewing Barley Grains
Source: Toxins (Basel). 2019 Jan 10;11(1):31. doi: 10.3390/toxins11010031 (PMC6357013; doi:10.3390/toxins11010031)
Supplement: Supplementary file 1 [file toxins-11-00031-s001.pdf]

Supplementary Materials: Assessment of toxigenic Fusarium species and their mycotoxins in brewing barley grains

Karim C. Piacentini, Liliana O. Rocha, Geovana D. Savi, Lorena Carnielli-Queiroz, Livia De Carvalho Fontes and Benedito Correa

Table S1. *Fusarium* species references used in this study for phylogenetic analysis.

**Accessionnumber**	***Fusariumspecies***	**NCBI accessionnumber**	
		EF-1α	*RPB2*
**NRRL26755**	*F.acaciae-mearnsii*	AF212449	KM361658
**NRRL29133**	*F.armeniacum*	HM744659	HQ154448
**NRRL31970**	*F.armeniacum*	HM744664	HQ154453
**NRRL6227**	*F.armeniacum*	HM744692	HQ154480
**NRRL13818**	*F.asiaticum*	AF212451.1	JX171573.1
**FRC R-09495**	*F.avenaceum*	GQ915502	GQ915486
**NRRL26916**	*F.boothii*	GQ915503.1	GQ915487
**FRC R-09624**	*F.cerealis*	GQ915505	GQ915489
**NRRL13721**	*F.cerealis*	AF212464	KM361656
**NRRL29297**	*F.cortaderiae*	AY225885.1	KM361662.1
**CBS139512**	*F.culmorum*	KT855186	KT855212
**FRC R-09618**	*F.culmorum*	GQ915506	GQ915490.1
**NRRL25475**	*F.culmorum*	AF212463	JX171628
**NRRL13402**	*F.equiseti*	JX171566.1	GQ505592.1
**NRRL20697**	*F.equiseti*	JX171595.1	GQ505594.1
**CBS128539**	*F.graminearum*	KT855182	KT855208
**CBS138561**	*F.graminearum*	KT855183	KT855209
**CBS138562**	*F.graminearum*	KT855184	KT855210
**CBS138563**	*F.graminearum*	KT855185	KT855211
**CBS139513**	*F.graminearum*	KT855187	KT855213
**CBS139514**	*F.graminearum*	KT855188	KT855214
**NRRL31084**	*F.graminearum*	HM744693	HQ154481
**NRRL26417**	*F.incarnatum-equiseti*	GQ505598	JX171635
**NRRL32175**	*F.incarnatum-equiseti*	GQ505609	JX171645
**NRRL53438**	*F.langsethiae*	HM744690	HQ154478
**NRRL53439**	*F.langsethiae*	HM744691	HQ154479
**NRRL28436**	*F.meridionale*	AF212435	KM361660
**NRRL25797**	*F.mesoamericanum*	AF212441	KM361657
**FRC T-0962**	*F.poae*	GQ915511	GQ915495
**NRRL28062**	*F.pseudograminearum*	AF212468	JX171637
**FRC R-07843**	*F.sambucinum*	GQ915512	GQ915496
**NRRL53421**	*F.sibiricum*	HM744675	HQ154463
**NRRL53422**	*F.sibiricum*	HM744676	HQ154464
**NRRL54149**[a]	*Fusarium*sp.	HM068337	HM068359
**NRRL13440**	*F.sporotrichioides*	HM744650	HQ154439
**NRRL3299**	*F.sporotrichioides*	HM744665	HQ154454
**NRRL25481**	*F.tricinctum*	HM068307	JX171629
**FRC R-09186**	*F.venenatum*	GQ915515	GQ915499

[a] *Fusarium* sp. NRRL54149: currently described as *Fusarium torreyae*(O Ɗonnell et al., 2013).

Table S2. Characteristics of the method performance for extraction of deoxynivalenol and zearalenone in barley grains and culture media.

Analytes	Matrix	LOD (μg/kg)	LOQ (μg/kg)	Linear regression (R^2)	Spiking level (μg/ml)	Recovery (%)	RSD (%)
DON	Barley grains	5	25	0.997	0.0375	98	6.4
					0.375	99	5.2
					0.500	99	3.3
	Fungi strains	5	25		0.025	98	5.7
					0.125	99	2.9
ZEA	Barley grains	10	25	0.999	0.0375	97	3.0
					0.375	98	2.3
					0.500	99	0.4
	Fungi strains	10	25		0.025	102	2.1
					0.125	98	1.5
